# Sarcomatous Tumor of the Breast Successfully Removed

**Published:** 1822-10

**Authors:** James Elliott

**Affiliations:** Member of the Royal College of Surgeons, London, and Surgeon to the Forces, Barbadoes.


Art. VII.—*Sarcomatous Tumor of the Breast successfully removed.* By James Elliott, Member of the Royal College of Surgeons, London, and Surgeon to the Forces, Barbadoes.

Mary Lambert, ætat. forty, a woman of colour, native of Antigua, but now residing at Barbadoes, consulted me in January last (1822,) respecting a disease of her right breast. On examining it, I found a large globular enlargement of the mammary gland, for the most part hard to the feel, but irregular and knotty on the upper part: one spot, a little distance from the nipple, was soft and of a blue colour, easily compressible, evidently showing that a fluid was contained within. The tumor was very moveable, and apparently not morbidly attached to the pectoral muscle; one of the glands in the axilla was enlarged, but not indurated. She complained of occasional lancinating pains, and much uneasiness in the affected part; was much incommoded by its weight, and was in a very anxious state of mind as to its consequences: in other respects her constitution was good, and the catamenia regular.

On inquiring into the cause, duration, &c. of the disease, she gave me the following history of her case:—She stated that, nearly nine years ago, she received an injury from a key of a door, which, while in the lock within, being violently pushed against her by the bursting open of the door from without, inflicted a severe blow on the right breast; she nearly fainted at the time from the effect of pain; the breast did not swell, but looked red and inflamed. This continued for some days, and then went off; no hardness remained. About two years afterwards, she was delivered of a female child, and while suckling it always felt pain in the breast, and not at any other time. The pain was not confined to any particular part, but generally throughout the gland. After nursing the child eighteen months, she weaned it: soon afterwards a small lump, not bigger than a hazle-nut, was perceived just above the nipple. It continued to increase gradually in size, but was not attended with any pain until twelve months afterwards, when it began to cause some uneasiness, and she felt occasional, but not severe, lancinating pains through the tumor, which had then become enlarged: it looked red and inflamed on the top, itched very much, and her continual scratching the part increased the irritation. The tumor, which then had attained the size of a tennis ball, still continued, during the course of years, gradually to increase in bulk; became also more painful and troublesome from its weight; until at length it acquired the state it was in when first seen by me.

Various remedies had been from time to time recommended by different medical men for its relief: some advised the removal of the breast, but never performed the operation; others deemed it as improper. When I first saw her, I endeavoured strongly to impress on her mind that any other treatment than the removal of the tumor by the knife was useless, and that, if the disease were allowed to go on, ulceration and bursting of it would finally ensue at the part which felt soft; and, probably, a large, open, foul sore would be produced, which, by its continual and profuse discharge, attended with great pain, would ultimately destroy her life; and that, when once such a state of the parts had been induced, the operation would be of no avail.

Notwithstanding I proposed the removal of the tumor as the only remedy that offered her a chance of getting rid of the disease, she did not at that time give her consent, but requested time to consider of it; and it was not until two months afterwards that I extirpated the tumor. For some days previous to the operation, the diseased breast became more painful, was hotter to the feel, and evidently showed a disposition to a change which would cause the tumor to open at the soft blue part; I therefore pressed the necessity of not delaying it any longer. The other breast was become tender and swollen; as was also the gland in the axilla, which was before affected; and there was likewise some general excitement in the system. Her menses were expected the following week: the operation, therefore, was performed under more unfavourable circumstances, from the tumor being in a state of irritation at the time.

On the 14th of March following, I removed the tumor, in the presence of several military surgeons. The patient being seated in a chair, and supported by assistants, I commenced the first incision near the axilla, and carried it along the side of the tumor above, in a line nearly transversely towards the sternum, that being its greatest diameter, saving as much integument as I considered prudent. It was my intention to have made a corresponding incision at the inferior part; but, as the patient seemed to suffer much from pain and terror, I continued to dissect behind the tumor, drawing it forward at the same time, until it was separated from its attachments beneath; I then, with a few strokes of the scalpel, divided the integuments below, saving as much as I deemed safe. The arteries were tied as they were divided; the external mammary was large, and bled in a full stream when divided, but was immediately secured. I was fortunate enough to succeed in the plan I had laid down in my own mind previous to the operation,—that of removing the whole tumor without penetrating its capsule with the knife, being aware that, had I done so, from the escape of its fluid contents, it would have collapsed in some degree, and have rendered the dissection more tedious and difficult. No portion of the diseased part was left behind; the cyst at a small spot adhered to the pectoral muscle, and, to remove it effectually, I dissected out the few fibres of that muscle to which it was attached.

After the tumor was removed, the patient fainted, rather from terror than loss of blood. Fortunately, the divided arterial branches were secured, so that the syncope was not increased or kept up by hemorrhage. Some wine and water was given her, and she recovered. The sides of the wound were brought in as close contact as the nature of the parts would allow, by straps of adhesive plaster, over which light dressings were applied, and the whole supported by a bandage. An anodyne was prescribed, and she was put to bed.

On examining the tumor after its removal, the disease appeared to be confined to the mammary gland, which was greatly enlarged. In the centre was an irregular cavity, the lining of which was smooth to the feel, containing about half a pint of dark bloody fluid; the whole contained in a cyst, or capsule, of condensed cellular substance. The cyst at the upper part near the nipple was in close contact with the external skin, both of which were so thin in one part, that the bloody fluid within imparted the blue colour before mentioned. On making a section of the diseased gland, it appeared of a whitish colour, rather firm to the feel, and in a morbid state of organization. The tumor appeared to be of the sarcomatous kind, often denominated schirrus; it weighed, including the fluid, about three pounds.

There were none of those white bands observed which are found to radiate from the centre of carcinomatous tumors to surrounding parts; nor was there, before its removal, any puckering of the external skin: the latter appeared somewhat stretched on the top, and thin; the knotty irregularity to the feel at that part arose from knobby portions of the diseased gland beneath.

I am disposed to consider the disease as local, and confined to the mammary gland: the soft enlargement of one of the glands in the axilla, the tenderness and swelling of the other breast, may be attributed to the extension of irritation. There was a good deal of fat around the base of the tumor, which did not appear to have partaken of any morbid action; of which I did not remove more than was lying in contact with the exterior of the diseased gland.

The wound, after a good deal of suppuration and discharge, healed in the course of a few weeks, leaving but a small cicatrix where granulations had formed. Four months have now nearly elapsed since the operation was performed: she is in very good health, following her usual occupations; the swelling of the gland in the axilla and of the other breast has subsided long since. She is entirely free from pain and uneasiness of any kind; there does not appear any sign whatever of a recurrence of the disease.*

*Barbadoes; June 29th,* 1822.

---

* We take this opportunity of thanking Mr. ELLIOTT for his communication, and trust that it will stimulate our medical readers in the Colonies to follow his laudable example, in transmitting the results of their observations and practice to us.—EDIT.

